# Synthesis, characterization and evaluation of anti-arthritic and anti-inflammatory potential of curcumin loaded chitosan nanoparticles

**DOI:** 10.1038/s41598-023-37152-7

**Published:** 2023-06-24

**Authors:** Hafiz Muhammad Asif, Farah Zafar, Khalil Ahmad, Amjad Iqbal, Ghazala Shaheen, Khalil Ahmad Ansari, Sehrish Rana, Rabia Zahid, Saira Ghaffar

**Affiliations:** 1 University College of Conventional Medicine, Faculty of Medicine and Allied Health Sciences, The Islamia University of Bahawalpur, Bahawalpur, Pakistan; 2 Department of Chemistry, University of Management and Technology, Lahore, Pakistan; 3 Department of Materials Technologies, Faculty of Materials Engineering, Silesian University of Technology, 44-100 Gliwice, Poland; 4 CEMMPRE- Centre for Mechanical Engineering Materials and Processes, Department of Mechanical Engineering, University of Coimbra, Rua Lui’s Reis Santos, 3030-788 Coimbra, Portugal

**Keywords:** Drug discovery, Health care, Medical research, Rheumatology, Nanoscience and technology

## Abstract

Curcumin is a phytochemical isolated from the dried rhizome of *Curcuma longa* L. family Zingiberaceae which possesses versatile biological activities and has hydrophobic properties. The current study was conducted to fabricate, and optimize curcumin loaded chitosan and Sodium tripolyphosphate (STPP) nanoparticles (NPs) to improve the bioavailability of curcumin. NPs were fabricated employing the Ionic gelation method. Four formulations were developed based on the selected variables like STPP and chitosan concentration, rotations per minute (rpm), temperature, and pH of chitosan solution. NPs were characterized for morphology, drug-polymer compatibility, yield, particle size, encapsulation efficiency, release behavior, anti-inflammatory and anti-arthritic activity compared to curcumin and standard drug. Fourier transform infrared spectroscopy (FTIR) shows nanoparticle compatibility. The maximum yield was 60%. Entrapment efficiency ranged from 45 to 65%. Curcumin NPs and standard drug 600 µg/ml shows 59% and 70% anti-inflammatory activity by HRB membrane stabilization method respectively which are greater than curcumin alone whereas anti-arthritic activity by protein denaturation method which is also comparable to standard drug and greater than curcumin was 66 and 70% respectively. Statistical analysis shows the mean significant difference at *p* ≤ 0.05. The study concluded that curcumin-loaded chitosan and STPP NPs formulated successfully by the Ionic gelation method, which increased curcumin absorption leading to a reduced dosing rate and improved patient compliance.

## Introduction

A collection of inflammatory disorders that influence tissues and joints are rheumatic disorders. The production of antibodies that acknowledge self-molecules that reside inside cells is a characteristic of those diseases. Such autoantibodies are formed as a loss in self-tolerance, and they cause inflammation and tissue damage in infected areas of the body^1^. Arthritis affects millions of people worldwide. The disease causes severe joint pain, stiffness, and swelling, which could result in disability if not managed effectively^2^. Rheumatoid arthritis (RA) is a severe autoimmune disorder that affects about 1% of the world's population and is associated with generous disability and mortality. RA is a chronic, long-lasting disease, where the failure to spontaneously relieve the inflammation allows the condition to persist in patients throughout their lives^3^.

Curcumin, a natural phenolic compound extracted from the rhizome of turmeric (*Curcuma longa* L.), belong to the family Zingiberaceae has generated research interest because it possesses pleiotropic biological and pharmacological properties, such as anti-cancer, anti-inflammatory, antibacterial, antioxidant and anti-rheumatic etc. The underlying mechanism includes its inhibitory effects on pro-inflammatory cytokines such as IL-6, TNF-α, IL-1β, cyclooxygenase (COX-2), inducible nitric oxide synthase (iNOS), and transcription factors such as nuclear factor (NF-kB), activator protein-1 (AP-1). However, the therapeutic efficacy of curcumin limited due to poor aqueous solubility (hydrophobic), rapid degradation (short half-life), and low bioavailability^4^. By encapsulating curcumin within chitosan nanoparticles, researchers aim to enhance its stability, improve its solubility, and prolong its release, leading to increased bioavailability and effectiveness.

Drug delivery systems (DDS) are technologies used to deliver pharmaceutical drugs to specific sites in the body to improve their efficacy, reduce toxicity, and enhance patient compliance. DDS has become a significant area of research in the pharmaceutical industry due to its ability to address various drug delivery challenges, including poor solubility, short half-life, and nonspecific distribution^5^. Nanoparticles are one of the most widely studied DDS. They can be designed to release drugs in a controlled manner, and they can target specific cells or tissues in the body. Recent studies have shown that nanoparticles can be used in the administration of a wide range of drugs, including anticancer drugs, antibiotics, and anti-inflammatory drugs. Examples of nanoparticles used in drug delivery include liposomes, dendrimers, and polymer nanoparticles^6^. NPs within range of 10–1000 nm are considered firm NPs or else particulate distribution. The active pharmaceutical ingredient in NPs is encapsulated by a polymer covering. NPs are categorized according to the type of polymers and the formulation process used ^7^. Polymeric NPs are extensively used in the treatment of many diseases due to their structure and flexibility. Chitosan is a polymer of nature and a polyelectrolyte of the cation. It has the possession of increasing in vivo as well as in vitro membrane permeability. It is degradable in serum and is biocompatible. Chitosan is Polysaccharide which has the potential to increase permeability and has mucoadhesive properties because of which it is used for increased absorption around the intestinal epithelium^8^. Sodium tripolyphosphate (STPP) is a commonly used reagent in the ionic gelation method for the synthesis of nanoparticles. STPP acts as a crosslinking agent that reacts with the cationic groups on the surface of the nanoparticles to form a stable nanoparticle matrix. Recent studies have reported the use of STPP in the synthesis of various types of nanoparticles, including silver nanoparticles (AgNPs), gold nanoparticles (AuNPs) and chitosan nanoparticles^9^.

Previously, some of the pharmacological studies like anti proliferative, wound healing properties, antioxidant, nephrotoxicity etc. of curcumin loaded chitosan nanoparticles have already been carried out and reported but the research on its anti-inflammatory and anti-arthritic activities are yet to be evaluated. Overall, the novelty of this research work lies in the combination of curcumin loaded chitosan NPs, anti-inflammatory as well as anti-arthritic activities. By exploiting the advantages of chitosan nanoparticles as drug carriers and investigating the potential improvements in anti-inflammatory properties, this research has the potential to contribute to the development of novel therapeutic strategies for inflammatory conditions. In our current work, we have fabricated and characterized curcumin loaded chitosan NPs with sodium tripolyphosphate (STPP) via Ionic gelation method to resolve the solubility problems. Anti-arthritic and anti-inflammatory properties by human red blood cell (HRB) membrane stabilization and protein denaturation method were also evaluated.

## Materials and methods

### Materials

Chitosan (Low molecular weight), Sodium tripolyphosphate, and curcumin were purchased from Sigma Aldrich, China. Sodium dihydrogen phosphate, ethanol (ethyl alcohol) and NaOH (Sodium hydroxide) were purchased from Merck Germany. Glacial acetic acid 100%, HCL (Hydrochloric acid) were purchased from Anala BDH Laboratory, England. Deionized water from industrial research lab, The Islamia University of Bahawalpur, Pakistan.

### Methods

#### Fabrication of curcumin loaded chitosan NPs by ionic gelation method

Curcumin loaded chitosan NPs were prepared with slight modification of previously reported ionic gelation method by Duse et al.^10^. Four formulations were prepared by varying chitosan and STPP concentration (5:0.5, 5.5:0.5, 6:0.5, 6:1) while stirring rate, temperature and pH remains constant as described in Table 1. Chitosan solution (2%) was prepared by adding it to 2 percent V/V glacial acetic acid solution on stirring at a pace of 500 rpm for 12 h on hot plate stirrer (ARE Heating Magnetic Stirrer). Two drops of tween 80 (polysorbate 80) was added, pH of chitosan solution was adjusted to 4.5 by adding 0.2 M NaOH and then filtered. Drug was dissolved in ethanol on constant stirring. After filtration, drug solution was added with the help of syringe at 600 rpm stirring. We prepared 0.1% solution of STPP (1 mg/ml) and added at 1000 rpm. Slightly turbid solution (Nano-suspension) was collected after 60 min, centrifuged at a speed of 12,000 rpm for 30 min. The supernatant was collected for further characterization whereas solid pellets of NPs were washed with deionized water and dried powder was collected after Lyophilization.

**Table 1:** Standard curve preparation of curcumin.

Dilution	Drug solution + Buffer (5 ml)	Final volume (ml)	Conc. (µg/ml)
1	5	10	25
2	5 of 1	10	12.5
3	5 of 2	10	6.25
4	5 of 3	10	3.125
5	5 of 4	10	1.562
6	5 of 5	10	0.781
7	5 of 6	10	0.3906
8	5 of 7	10	0.195
9	5 of 8	10	0.048
10	5 of 9	10	0.024
11	5 of 10	10	0.012
12	0 ml	10	0

### Characterization of curcumin NPs

#### Calibration of standard curve

Diverse dosages of curcumin (0 µg/ml to 25 µg/ml) dilutions were developed by mixing the stock solution with Hydrochloric acid Buffer 1.2 pH, phosphate buffer 6.8 and 7.4 separately as described in Table 1. Blank (HCl buffer, phosphate buffer 6.8 and 7.4) was adjusted to auto zero for measuring UV absorbance. The absorbance of curcumin in various dilutions was measured at 263 nm, and the graph was drawn by taking absorbance against concentration using Microsoft Excel. Data is given in Table 1. The graph of standard curve of curcumin in phosphate buffer (pH 6.8) has been provided in supplementary file.

### Yield analysis

The percentage yield was calculated as the weight of obtained dried NPs divided by the sum of weight of dried substances used initially, multiplied by a hundred, as expressed mathematically^11^.

$$Percent\;yield = \frac{{{\text{Actual}}\;{\text{amount}}\;{\text{of}}\;{\text{NPs}}\;{\text{obtained }}\left( {{\text{grams}}} \right){ }}}{{Theoretical\;amout \left( {{\text{grams}}} \right)}} \times 100$$

### Determination of entrapment efficiency (EE)

The capability of EE of drug in all the prepared NPs was measured using an indirect approach. Freshly produced formulations of NPs were mounted in centrifuge machines (Himac CS150GXL, Hitachi, Japan), and rpm was set for 35 min at 12,000. NPs were isolated after 35 min, and Supernatant was obtained to determine the quantity of unentrapped drugs using the following equation:

$$Total\;Amount\;of\;Loaded\;Drug = Total\;Amount\;of\;Drug - Amount\;of\;un\;entrapped\;drug$$

$$Entrapment\;Efficiency \left( \% \right) = \frac{{{\text{Actual}}\;{\text{drug}}\;{\text{content}}\;{\text{in}}\;{\text{NPs }}}}{Theoretical\;drug\;in\;Nanoparticles} \times 100$$

### Size and zeta-potential analysis

Size distribution and the average size of the chitosan NPs were measured, and the polydispersity index (PDI) was calculated. Size and size distribution investigations were conducted by DLS, using scattered light at 90° or 173° angles, automatically selected by the apparatus. The zeta potential measurement was done at 25 °C to investigate the size, dispersion and stability of chitosan NPs.

### Scanning electron microscopy (SEM)

After the preparation of chitosan NPs, the characterization of the nanoparticle was examined by SEM using ZEISS LEO SUPRA 55, field emission scanning electron microscope (FESEM).

### Fourier transform infrared spectroscopy (FTIR)

FTIR Spectroscopy is commonly used technique for molecule structural analysis, for determining chemical bonds between molecules, and for defining chemical structure. Specific functional groups found in the molecular chemical structure are resolute across the FTIR spectra absorption band. In the NPs Interaction between different substances and medicine, was noted. The FTIR spectra of the chitosan NPs sample were determined using Tensor 27 series FTIR.

### Release of drug (in vitro)

The drug-release activity was tested using paddle-type USP dissolution apparatus on curcumin loaded chitosan NPs. Weighted volumes of 5 mg equivalent NPs were spread in phosphate buffer 900 ml (pH 6.8). Stirred at 100 rpm, it preserved the dissolution medium at 37 ± 5 °C^12^. During the dissolution analysis of 15 h, 5 ml aliquots were removed from the dissolution medium at set intervals of time. Upon extraction of aliquots, 5 ml of fresh buffer was applied to preserve sink capacity. Collected sample spectrophotometrically assayed using UV-spectrophotometer (IRMACO GmbH, Geesthacht, Germany) at 263 nm. Percent Cumulative Release of drugs was drawn adjacent to time, representing the discharge behavior graphically^13^.

### In vitro anti-arthritic activity

The in vitro anti-arthritic activity was performed using egg albumin protein denaturation method previously reported by Abbas et al.^14^ with slight modifications. We used 2 ml of several concentrations (200, 400, 600 μg/ml) of curcumin, nanoparticle formulation (dissolve in ethanol), and standard drug diclofenac sodium (injection form), egg albumin 0.2 mL as well as phosphate buffer 2.8 mL (pH = 6.5). At 37 °C reaction mixture was incubated for 20 min followed by heating at 70 °C for 5 min. Absorbance was taken at 660 nm with a spectrophotometer after cooling reaction mixture to room temperature. Same quantity of egg albumin, Phosphate buffer was used as the negative control with 2 ml distill water and % inhibition was calculated by following formula:

$$\% {\text{Inhibition}} = {1}00 \times \left( {{\text{A}}_{{\text{C}}} - {\text{A}}_{{\text{S}}} } \right)/{\text{A}}_{{\text{C}}}$$

where AC = absorption of the control sample, AS = absorption of the test sample.

### In vitro anti-inflammatory activity by human RBCs (HRBC) membrane stabilization method

The HRBC membrane stabilization method was used for study of anti-inflammatory activity previously reported by Abbas et al.^14^ with slight modifications. Informed consent was obtained from all subjects, IFBDO (International Federation of Blood Donor Organizations) guidelines was adopted in the study, The study was approved by ethical Committee, The Islamia University of Bahawalpur. The blood sample was taken and mixed with equal volume of Alsever’s solution (0.8% Sodium citrate, 2% dextrose, 0.5% citric acid and 0.42% sodium chloride) and centrifuged at 3000 rpm. Isosaline was used to wash the packed cells, and a 10% v/v suspension was prepared. Here, 1 ml of several concentrations (200, 400 and 600 µg/ml) of curcumin, nanoparticle formulation (dissolve in ethanol) and same concentrations of standard drug diclofenac sodium (injection form) using distilled water were taken and to each concentration, 2 ml hypo saline, 1 ml of phosphate buffer and 0.5 ml of HRBC suspension were mixed. It was incubated at 37 °C for 30 min and centrifuged at 3000 rpm for about 20 min. The hemoglobin content of the supernatant solution was measured at 560 nm spectrophotometrically.

The % inhibition was determined using the following equation:

$$\% {\text{Inhibition}} = {1}00 \times \left( {{\text{A}}_{{\text{C}}} {-}{\text{A}}_{{\text{S}}} } \right)/{\text{A}}_{{\text{C}}}$$

where A_C_ = control absorbance, A_S_ = test sample absorbance.

### Statistical analysis

One way ANOVA followed by LSD post hoc test was applied for checking statistical significance. The significance was determined at *p* ≤ 0.05. The results were analyzed by using IBM SPSS statistics 20 version.

## Results and discussion

### Preparation of curcumin loaded chitosan NPs

The chitosan-based, polymeric NPs of curcumin were prepared by Ionic gelation method. Total four formulations were prepared. Table 2 provides a set of formulations and several variables. In the current study, curcumin loaded NPs were prepared, which exhibited more significant activity than curcumin. The reduced particle size and positive zeta potential exhibited sustained release of drug at diseased site, it increased absorption which is expected mechanism to improve its anti-inflammatory effect. Hence proven that NPs improved the bioavailability, solubility, and stability of curcumin. NPs were synthesized and characterized successfully with varying stirring speed, polymer, and surfactant concentrations.

**Table 2:** Formulations with different variables.

Formulation code	Drug (mg)	Chitosan (mg)	Sodium Tripolyphosphate(mg)	Tween 80 (Drops)
CCNF1	1	5	0.5	1
CCNF2	1	5.5	0.5	2
CCNF3	1	6	0.5	2
CCNF4	1	6	1	2

### Average particle size and morphology

**Figure 1:**
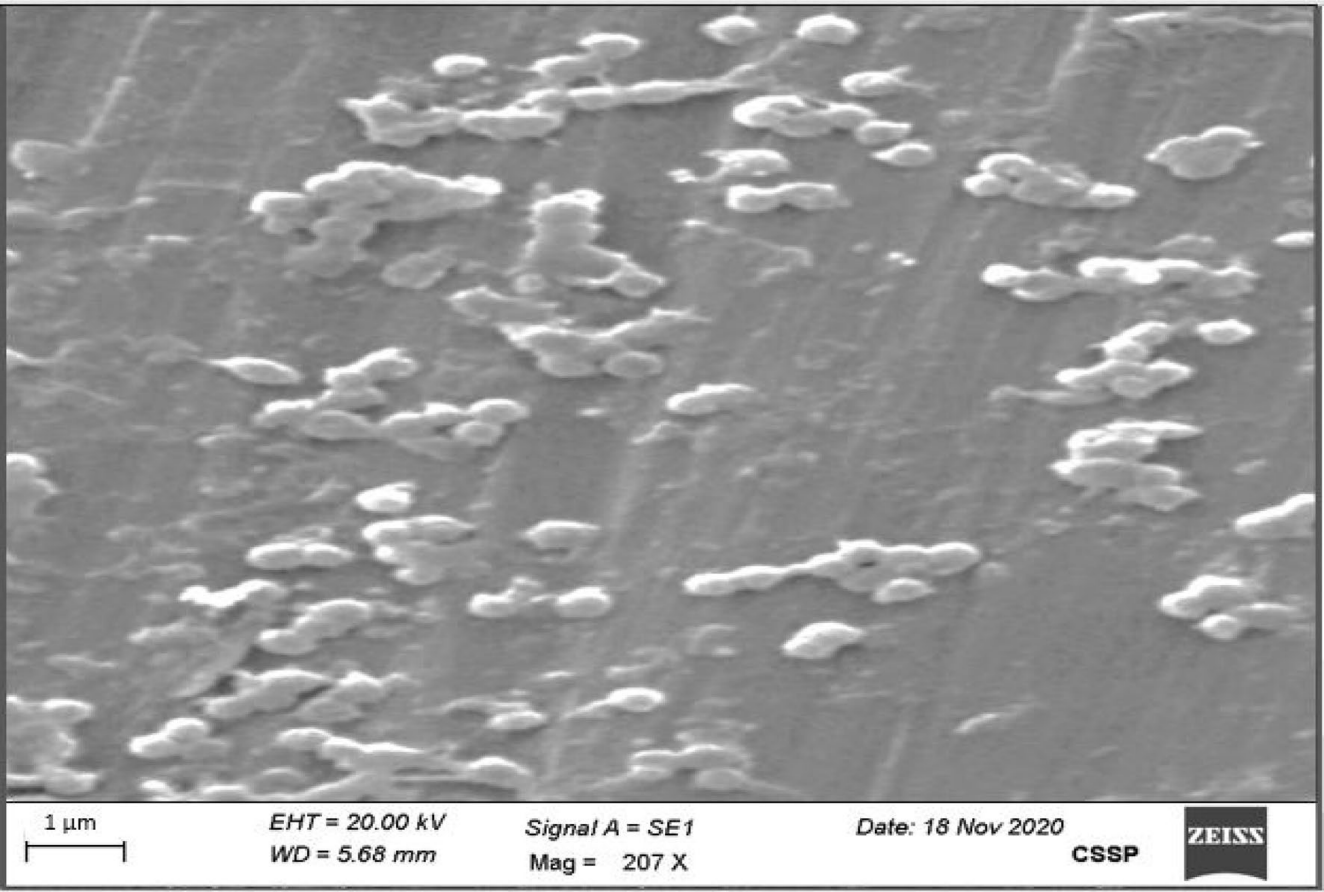
SEM image of chitosan-curcumin NPs.

The DLS instrument was used to measure the particle size, PDI, and zeta potential of NPs. All formulations of NPs have average sizes given below in Table 3. The average diameter of CCNF1 was 481.7 and PDI was 0.793. Zeta potential was 37.7 mV. Several parameters such as shape, size, composition, and size distribution, could have a prominent effect on the anti-arthritic activity of chitosan NPs. Therefore, the shape and size of chitosan NPs were characterized by SEM. Figure 1 shows the SEM image of the synthesized chitosan NPs at resolution of 1 µm at voltage 20 kV and synthesized chitosan NPs have spherical shape. Optimized formulation with smallest size was formulation CCNF1 which contains chitosan and STPP ratio i.e., 5:0.5, surfactant concentration of 1%, and stirring speed 800 rpm. All the formulations have a particle size within range 1–1000 nm. Largest particle size was of formulation CCNF4 which has chitosan and STPP ratio of 5:1. CCNF1 Nanoparticles size analysis by zeta potential and Zeta potential graph of nanoparticle formulation CCNF1 has been provided in supplementary file. In a previous study conducted by Sruthi Sreekumar et al. in 2018 reported that the average hydrodynamic diameter of the particles generated depends significantly on the initial chitosan concentration for a given chitosan to STPP molar ratio^15^. The second level of acetylation of the chitosan was discovered most significant element affecting the system's capacity to produce particles. It's interesting to note that viscosimeter investigations revealed that the presence or absence of salts in the medium had a significant impact on particle production and the average hydrodynamic diameter of the particles created. The second level of acetylation of the chitosan was discovered. In conclusion, They observed that it is possible to control production and characteristics of chitosan particles ranging in size from nano- to micrometers by manipulating two factors of the polymer solution, namely its initial concentration and its solvent environment^16^.

**Table 3:** Average particle size of curcumin chitosan NPs.

Formulation code	Average particle size (nm)	Standard deviation	PDI
CCNF1	481.7	481.70 ± 8.06^a^	0.793
CCNF2	580.6	580.60 ± 10.51^b^	0.816
CCNF3	656.5	656.50 ± 7.81^c^	0.874
CCNF4	761.5	761.50 ± 13.01^d^	1.00

“a” showed that size significantly low than b, c and d.

### Percentage yield and entrapment efficiency (EE)

Formulations (CCNF1-4) percentage yield was found in range of 25 to 60% as well as EE 45–65% as shown in Table 4. The maximum percentage yield was found in formulation CCNF4. The percentage EE and yield of the optimized CCNF1 formulation were 45%, and 25%, respectively. The mass ratio of chitosan to STPP (Chitosan: STPP) directly affected percentage yield, percentage entrapment and percentage drug load. In a study conducted by Akolade et al., 2018 showed the same result as STPP concentration increases as compared to chitosan, a yield of NPs also increases^17^. Less percentage yield is due to the chemical interaction between the phosphate group of STPP anion and the hydroxyl group of phytochemicals. Because of this competition, STPP anions have less contact with chitosan molecules resulting in a low percentage yield^18^, which is produced by CCNF1 in the current study. In our study NPs shows a spherical shape which is confirmed by a previous study conducted by Ma et al., 2020, it was shown that the curcumin along with chitosan possess spherical morphology and compact structure when observe under SEM^19^.

**Table 4:** Formulations percentage yield and entrapment efficiency.

Formulation Codes	Percentage yield	Percentage entrapment efficiency
CCNF1	25	45
CCNF2	30	50
CCNF3	45	55
CCNF4	60	65

### Fourier transform infrared spectroscopy (FTIR)

**Figure 2:**
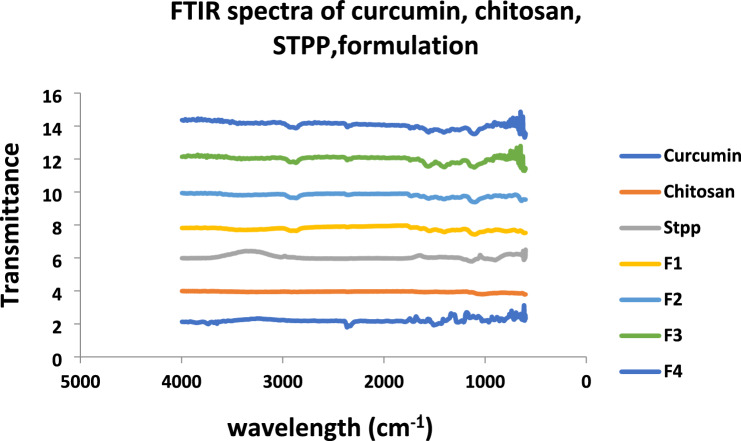
FTIR spectrum of curcumin, chitosan, STPP, nanoparticle formulations CCNF1-4.

The FTIR transmittance spectra of polymers, crude drugs, and NPs formulations were analyzed on the tensor 27 series FTIR. The wavelength range of spectra was 500 to 4000 cm^−1^. FTIR analyses were done at a resolution of 4 cm^-1^ to elaborate the potential functional groups in plant extracts that are responsible for capping the formed chitosan NPs. A peak at 3519 cm^−1^ indicates the O–H group. The appearance of FTIR peak at 2932.6 cm^−1^ and 2880.4 cm^−1^(for CH_3_ and CH_2_ groups respectively) indicate Hydrocarbons, while the small peak shows Halogens (as C–Cl stretches at 1160.1 cm^−1^), Ethers (as C–O stretch at 1160.1 cm^−1^) and bending vibration of –CH bond of alkene group at peaks 728 cm^−1^ and 950 cm^−1^. Shown in Fig. 5 and chitosan peaks in Fig. 2. The entire functional groups peaks are shown in NPs formulations from FTIR data analysis and due to interaction between amino groups of chitosan and STPP phosphate groups it is confirmed that NPs are fabricated which is also shown in previous study conducted by khan et al. in 2016 that drug is loaded in chitosan because of interaction among amino group of chitosan and keto group of curcumin^20^. The separate FTIR spectra of curcumin, chitosan, STPP, F1, F2, F3 and F4 have been provided in supplementary file.

### In vitro NPs drug release study

**Figure 3:**
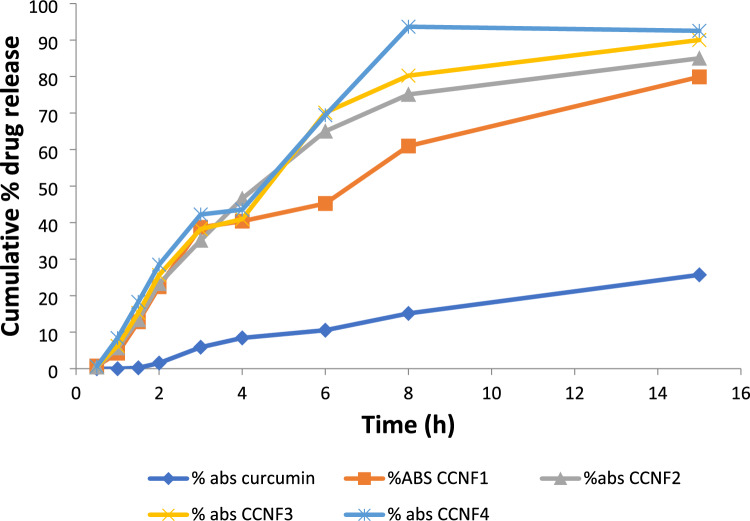
Combine cumulative % drug release of curcumin and CCNF1-4 in 6.8 pH phosphate buffer.

After 15 h, the release rate of drug from NPs of all formulations (F1–F4) in percentage was observed from samples absorbance and was found in the range of 60% to 83% whereas pure drug cumulative release was 25%. The cumulative release percent of pure drug and formulations is demonstrated in the Table 5 and graph shown in Fig. 3. By performing dissolution studies drug releases from NPs of curcumin result in increased bioavailability of drug. After 15 h, release rate of drug from NPs of all formulations (CCNF1-CCNF4) in percentage was observed from samples absorbance and was found in the range of 60% to 83% whereas curcumin cumulative release was 25%^15^. Curcumin is less soluble and has low bioavailability which can be increased by encapsulation of curcumin as formation of NPs which is also shown by a previous study conducted by Madhvi et al.^21^.

**Table 5:** In vitro cumulative release % of curcumin and CCNF1-4 formulations.

Time (hour)	Drug	CCNF1	CCNF2	CCNF3	CCNF4
0	0	0	0	0	0
1	0	0.74	0.45	0.56	0.60
1.5	0.24	4.25	5.68	6.25	8.39
2	1.54	12.85	13.39	15.29	18.31
3	5.88	22.45	23.28	25.65	28.49
4	8.43	38.71	40.15	38.59	42.33
6	10.54	40.39	46.59	40.97	43.55
8	15.14	45.24	65.02	70.12	69.41
15	25.7	60.97	75.10	80.25	83.65

### In vitro anti-arthritic activity by protein denaturation method

**Figure 4:**
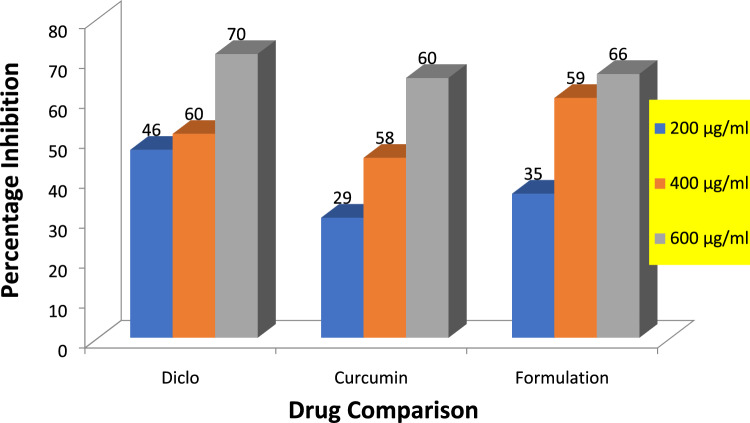
Graphical representation of percentage inhibition of diclofenac sodium, curcumin, and formulation by protein denaturation method.

By protein denaturation method, percentage absorption of 200, 400, and 600 µg/ml of curcumin and nanoparticle formulation is (29, 58, 60%) and (35, 59, 66%) respectively compared to standard drug i.e., diclofenac sodium having percentage inhibition (46, 60, 70%) for the same concentrations respectively as shown in Table 6 and graph shown in Fig. 4.

**Table 6:** Percentage absorption and inhibition by protein denaturation method.

Concentration µg/ml	Percentage absorption %	Percentage protection%
Curcumin		
200	0.748	29.0 ± 1.0*
400	0.433	58.6 ± 0.5*
600	0.311	60.6 ± 0.5
Formulation		
200	0.688	35.6 ± 0.5*
400	0.427	59 ± 0.5*
600	0.358	66.0 ± 1.0*
Diclofenac sodium		
200	0.519	46.6 ± 0.5*
400	0.565	60.6 ± 0.5*
600	0.303	70.6 ± 0.5

*The mean difference is significant at *p* ≤ 0.05 level. One way ANOVA followed by LSD post hoc test was applied for checking statistical significance.

### In vitro anti-inflammatory activity by the process of human red blood cell (HRBC) membrane stabilization

**Figure 5:**
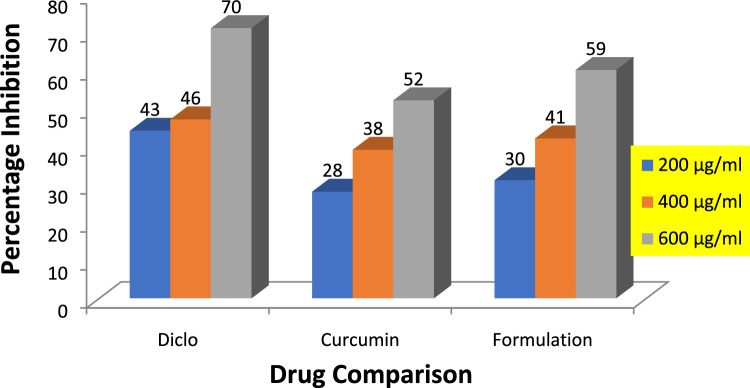
Graphical representation of percentage inhibition of diclofenac sodium, curcumin, and formulation by anti-inflammatory activity.

By the HRBC membrane stabilization method, percentage protection of 200, 400, and 600 µg/ml of curcumin and Nanoparticle is (28, 38, 52%) and (30, 41, 59%) respectively as compared to standard drug i.e., diclofenac sodium having percentage inhibition (43, 46, 70%) for the same concentrations respectively as shown in Table 7 and graph shown in Fig. 5. Previous studies reported that compounds with membrane stabilizing properties have the ability to interfere with phospholipases release, which triggers inflammatory mediators formation, several plants with anti-inflammatory properties can inhibit protein denaturation which is induced thermally^22^.

**Table 7:** Percentage absorption and inhibition of curcumin, formulation, and diclofenac sodium by HRBC membrane stabilization method.

Concentration µg/ml	Percentage absorption %	Percentage protection %
Curcumin		
200	0.037	28 ± 0.5*
400	0.023	38.6 ± 0.5*
600	0.018	52.0 ± 1.0*
Formulation		
200	0.026	30.3 ± 0.5*
400	0.022	41.6 ± 0.5*
600	0.015	59.6 ± 0.5*
Diclofenac sodium		
200	0.021	43.6 ± 0.5*
400	0.020	46.0 ± 1.0*
600	0.011	70.6 ± 0.5*

*The mean difference is significant at *p* ≤ 0.05 level. One way ANOVA followed by LSD post hoc test was applied for checking statistical significance.

## Conclusion

It is concluded from the current study that encapsulation of curcumin within NPs brought a new way to improve curcumin solubility and bioavailability. Furthermore, these NPs possess marvelous in vitro anti-inflammatory and anti-arthritic activities by human red blood cell membrane stabilization and protein denaturation methods respectively. In vitro drug dissolution study showed that the curcumin bioavailability enhanced from nanoparticles, that’s why efficacy is improved and dosage frequency can also be reduced. In vivo studies and clinical trials are necessary to prove its activity and interpret anti-inflammatory mechanism of action.

## Supplementary Information

Supplementary Information.

## Data Availability

The data that support the findings of this study are available from the corresponding author, upon reasonable request.

## References

[1] Apel F, Zychlinsky A, Kenny EF (2018). The role of neutrophil extracellular traps in rheumatic diseases. Nat. Rev. Rheumatol..

[2] Said MS, Hashmi AM, Hussain A (2022). Clinical evaluation of patients suffering from osteoarthritis along with prevalence, pharmacological and non-pharmacological treatment. Int. J. Nat. Med. Health Sci..

[3] Chen Z, Bozec A, Ramming A (2019). Anti-inflammatory and immune-regulatory cytokines in rheumatoid arthritis. Nat. Rev. Rheumatol..

[4] Prado-Audelo D, María L, Caballero-Florán IH (2019). Formulations of curcumin nanoparticles for brain diseases. Biomolecules.

[5] Katouzian I, Taheri RA (2021). Preparation, characterization and release behavior of chitosan-coated nanoliposomes (chitosomes) containing olive leaf extract optimized by response surface methodology. J. Food Sci. Technol..

[6] Khan MS, Asif MI, Asif M (2022). Fabrication and characterization of curcumin loaded ZnO nanoparticles and their in vitro antibacterial activity. Int. J. Nat. Med. Health Sci..

[7] Robertson JD, Rizzello L, Avila-Olias M (2016). Purification of nanoparticles by size and shape. Sci. Rep..

[8] Hadidi M, Pouramin S, Adinepour F (2020). Chitosan nanoparticles loaded with clove essential oil: Characterization, antioxidant and antibacterial activities. Carbohyd. Polym..

[9] Poureghbal Y, Rahimi M, Akbari M (2022). Ionic gelation of chitosan with sodium tripolyphosphate using a novel combined nebulizer and falling film system. Can. J. Chem. Eng..

[10] Duse L, Baghdan E, Pinnapireddy SR (2018). Preparation and characterization of curcumin loaded chitosan nanoparticles for photodynamic therapy. Phys. Status Solidi.

[11] Bhardwaj P, Chaurasia H, Chaurasia D (2010). Formulation and in-vitro evaluation of floating microballoons of indomethacin. Acta Pol. Pharm..

[12] Deshmukh RK, Naik JB (2013). Diclofenac sodium-loaded Eudragit® microspheres: Optimization using statistical experimental design. J. Pharm. Innov..

[13] Nath B, Nath LK, Kumar P (2011). Preparation and in vitro dissolution profile of zidovudine loaded microspheres made of Eudragit RS 100, RL 100 and their combinations. Acta Pol. Pharm..

[14] Abbas MW, Hussain M, Qamar M (2021). Antioxidant and anti-inflammatory effects of Peganum harmala extracts: An in vitro and in vivo study. Molecules.

[15] Pontes-Quero GM, Benito-Garzón L, Cano JP (2021). Amphiphilic polymeric nanoparticles encapsulating curcumin: Antioxidant, anti-inflammatory and biocompatibility studies. Mater. Sci. Eng., C.

[16] Sreekumar S, Goycoolea FM, Moerschbacher BM (2018). Parameters influencing the size of chitosan-TPP nano-and microparticles. Sci. Rep..

[17] Akolade JO, Oloyede HOB, Salawu MO (2018). Influence of formulation parameters on encapsulation and release characteristics of curcumin loaded in chitosan-based drug delivery carriers. J. Drug Deliv. Sci. Technol..

[18] Dudhani AR, Kosaraju SL (2010). Bioadhesive chitosan nanoparticles: Preparation and characterization. Carbohyd. Polym..

[19] Ma S, Moser D, Han F (2020). Preparation and antibiofilm studies of curcumin loaded chitosan nanoparticles against polymicrobial biofilms of Candida albicans and Staphylococcus aureus. Carbohyd. Polym..

[20] Khan MA, Zafaryab M, Mehdi SH (2016). Characterization and anti-proliferative activity of curcumin loaded chitosan nanoparticles in cervical cancer. Int. J. Biol. Macromol..

[21] Madhavi M, Madhavi K, Jithan A (2012). Preparation and in vitro/in vivo characterization of curcumin microspheres intended to treat colon cancer. J. Pharm. Bioallied Sci..

[22] Rahman H, Eswaraiah MC, Dutta A (2015). In-vitro anti-inflammatory and anti-arthritic activity of *Oryza Sativa* Var. joha rice (an aromatic indigenous rice of Assam). Am. Eurasian J. Agric. Environ. Sci..

