# Supplementary material for: Synthesis, characterization and evaluation of anti-arthritic and anti-inflammatory potential of curcumin loaded chitosan nanoparticles
Source: Sci Rep. 2023 Jun 24;13:10274. doi: 10.1038/s41598-023-37152-7 (PMC10290721; doi:10.1038/s41598-023-37152-7)
Supplement: Supplementary file 1 — Supplementary Information. [file 41598_2023_37152_MOESM1_ESM.docx]

**Standard curve of Curcumin in Phosphate buffer (pH 6.8)**

The standard curve of curcumin in phosphate buffer is show in Figure 1.

**Figure 1.** Standard curve of curcumin in phosphate buffer (pH 6.8).


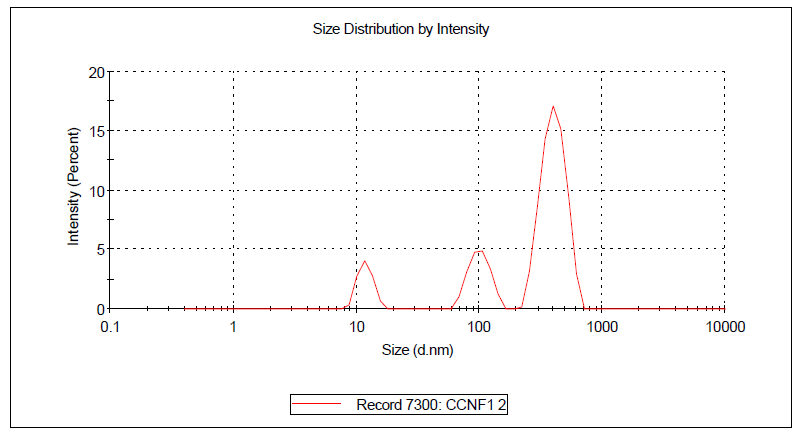


**Figure 2.** CCNF1 Nanoparticles size analysis by zeta potential.


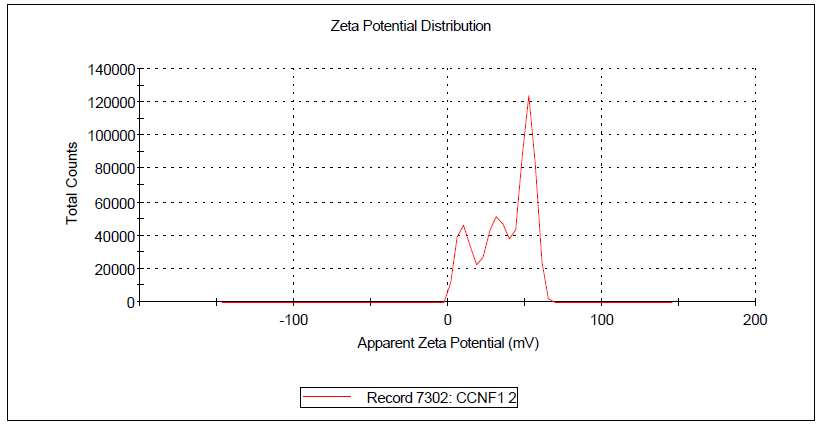


**Figure 3.** Zeta potential graph of nanoparticle formulation CCNF1.

FTIR graph


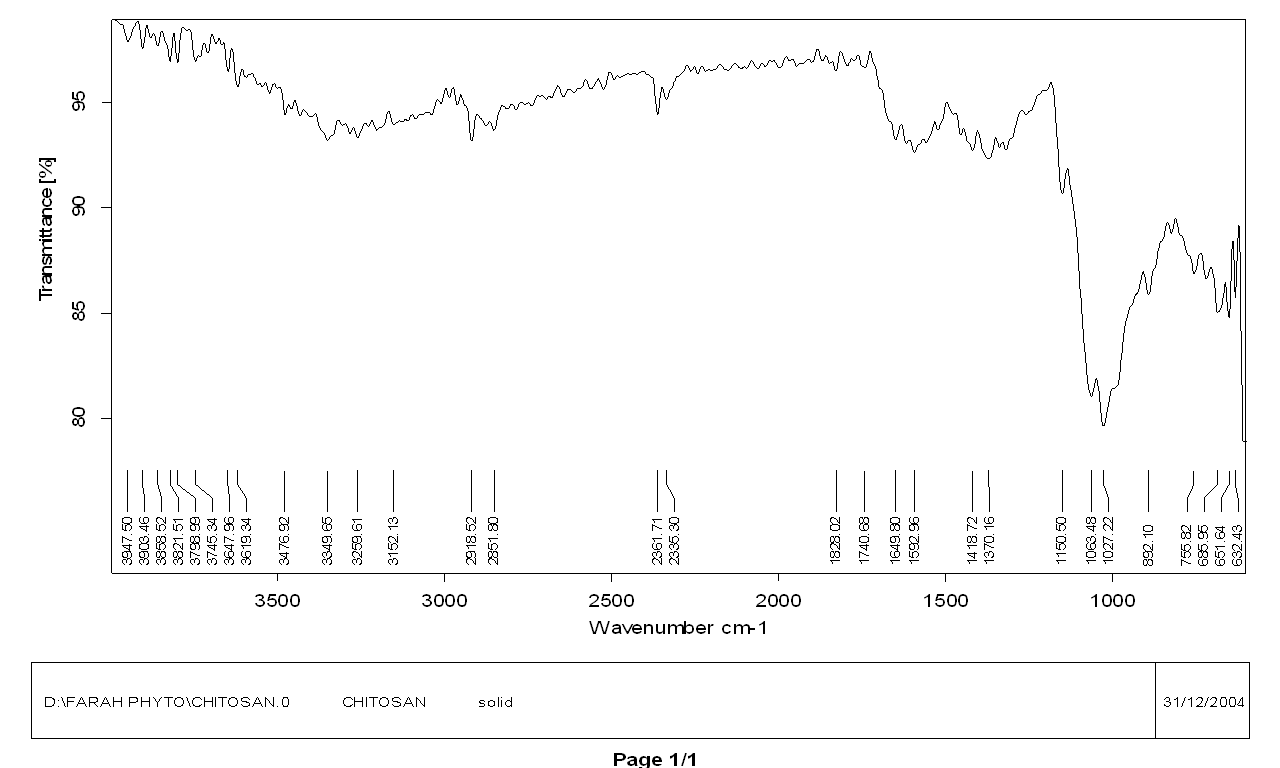


**Figure 4.** FTIR graph of chitosan.


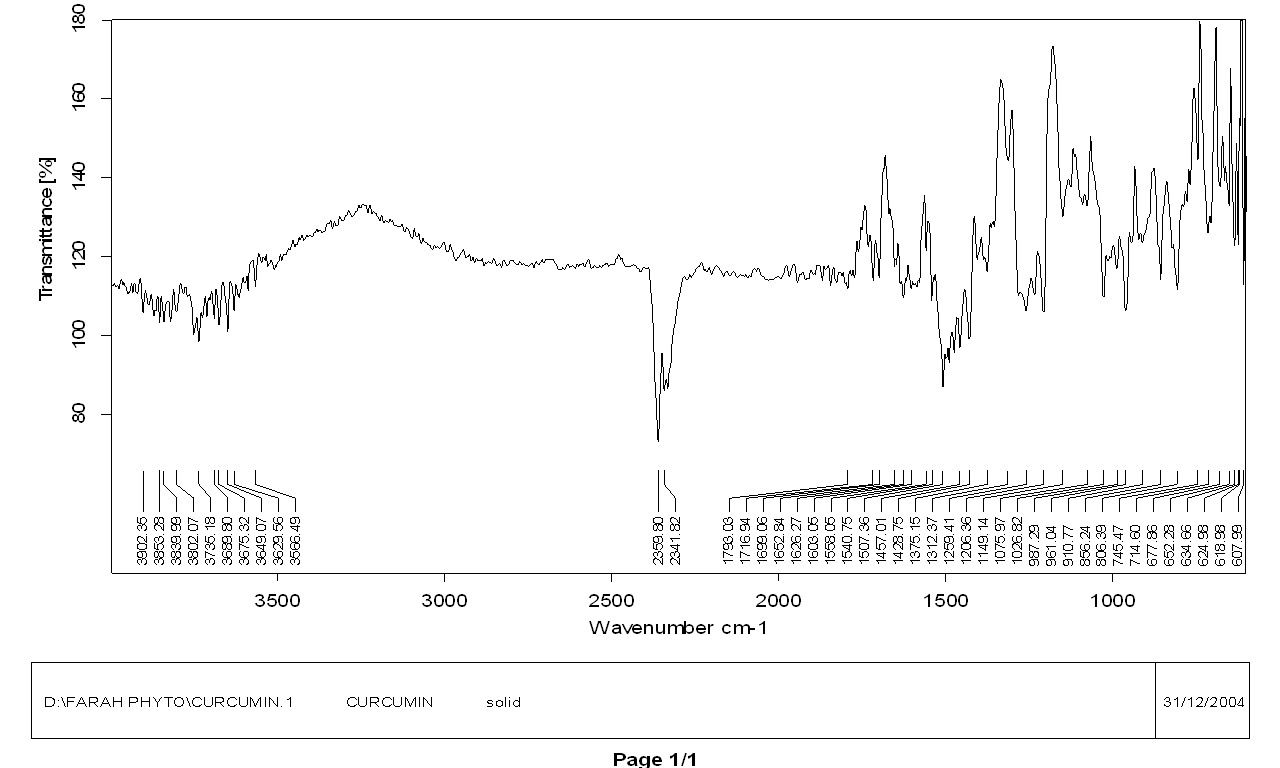


**Figure 5.** FTIR graph of curcumin.


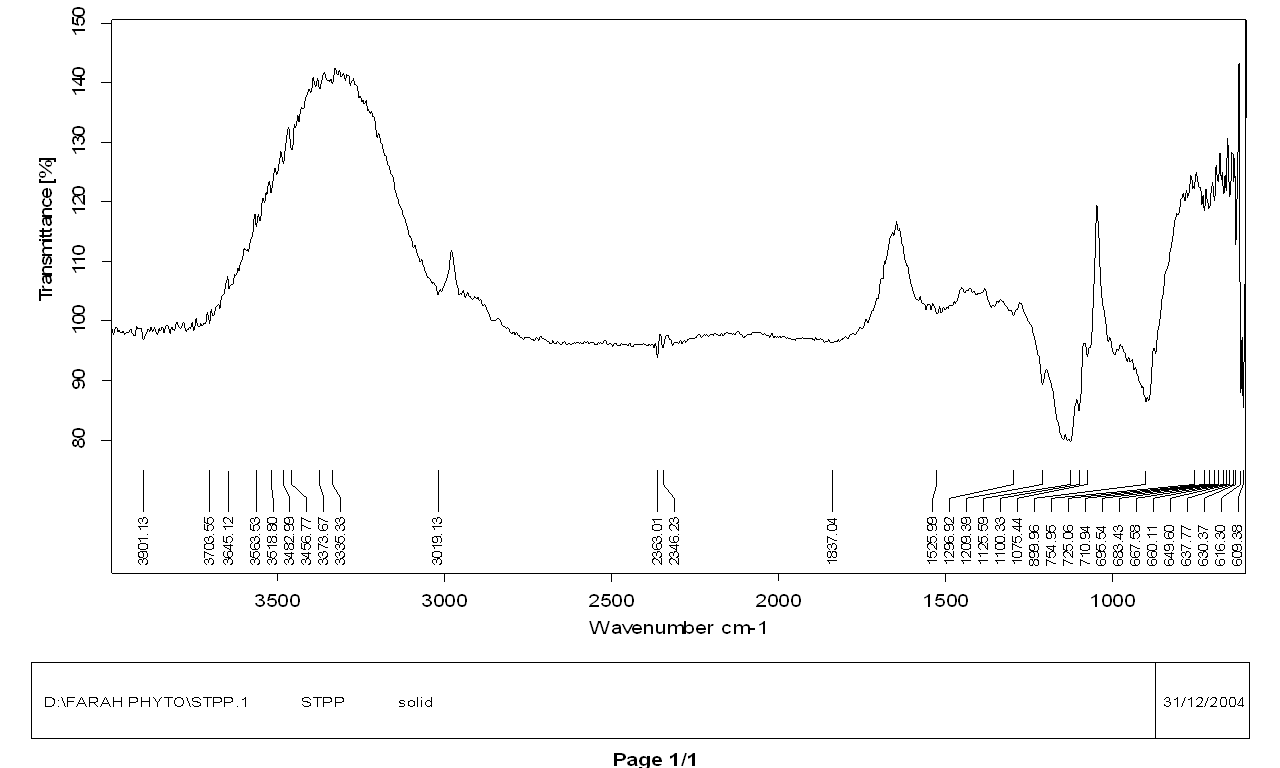


**Figure 6.** FTIR graph of STPP.


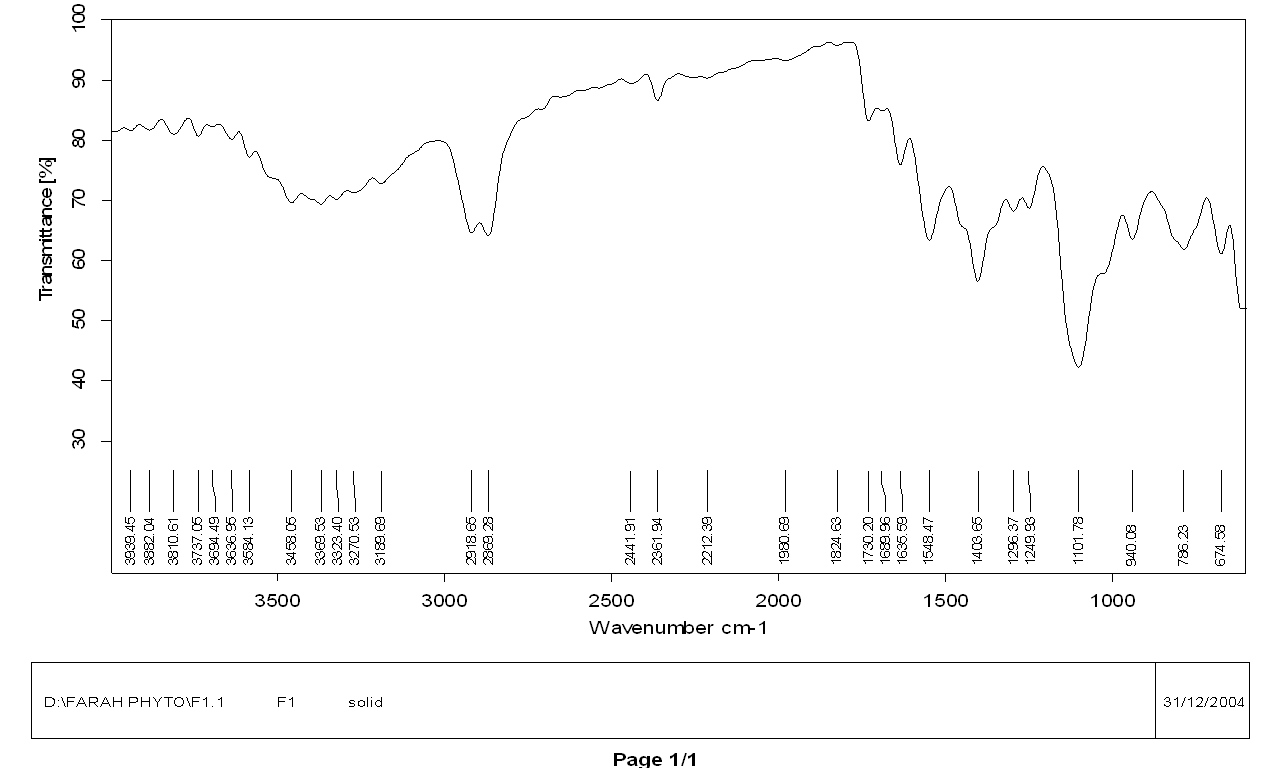


**Figure 7.** FTIR graph of F1.


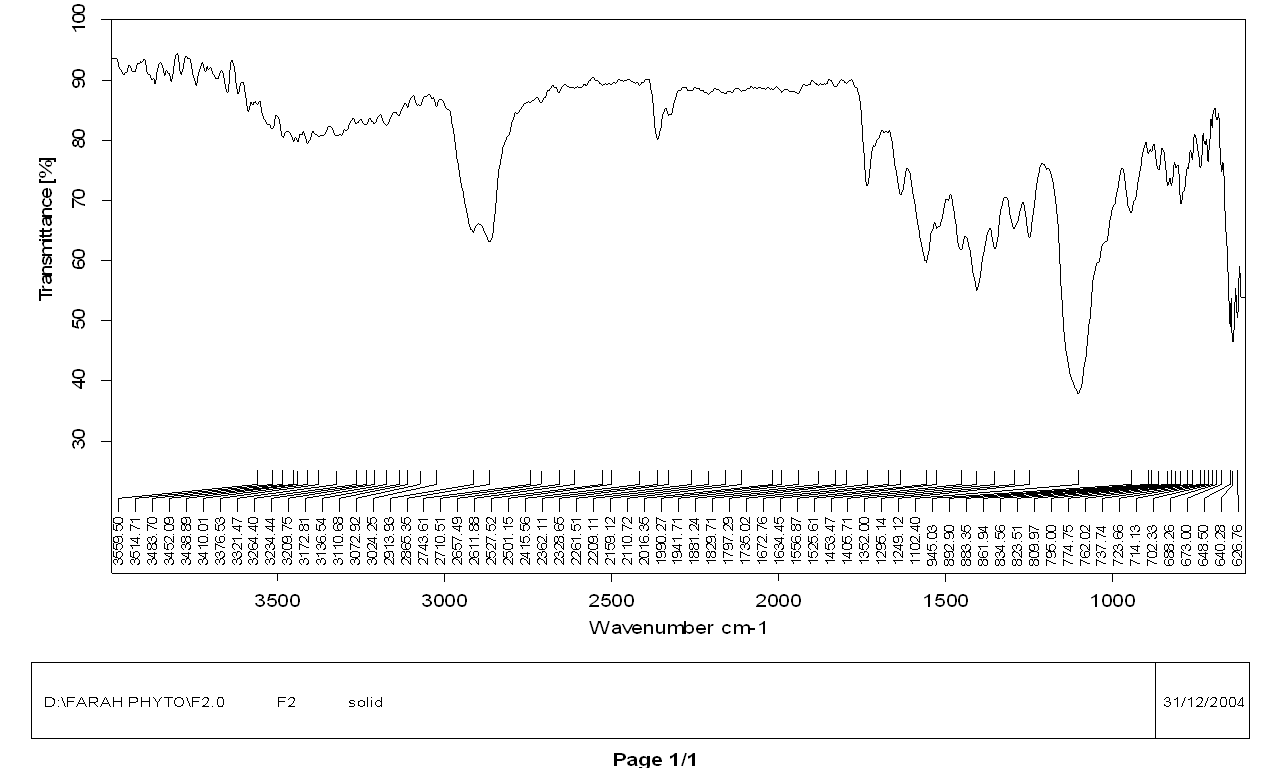


**Figure 8.** FTIR graph of F2.


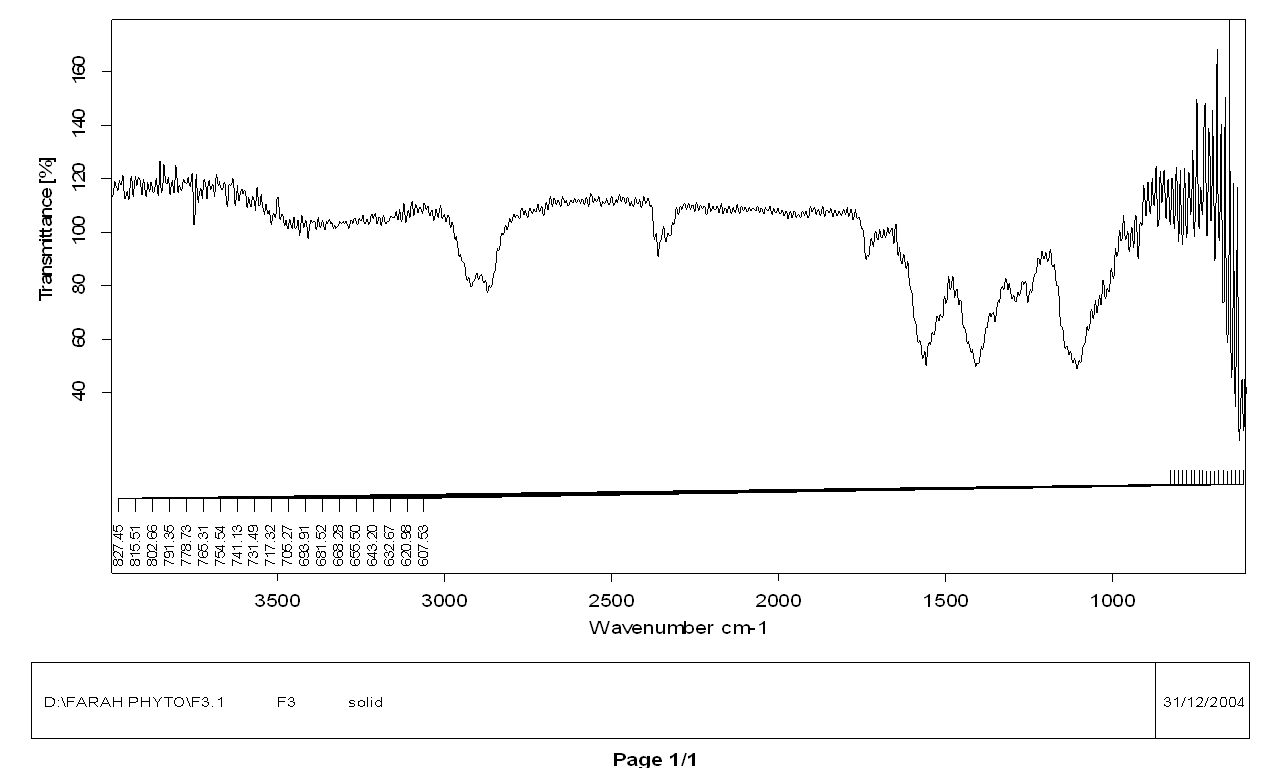


**Figure 9.** FTIR graph of F3.


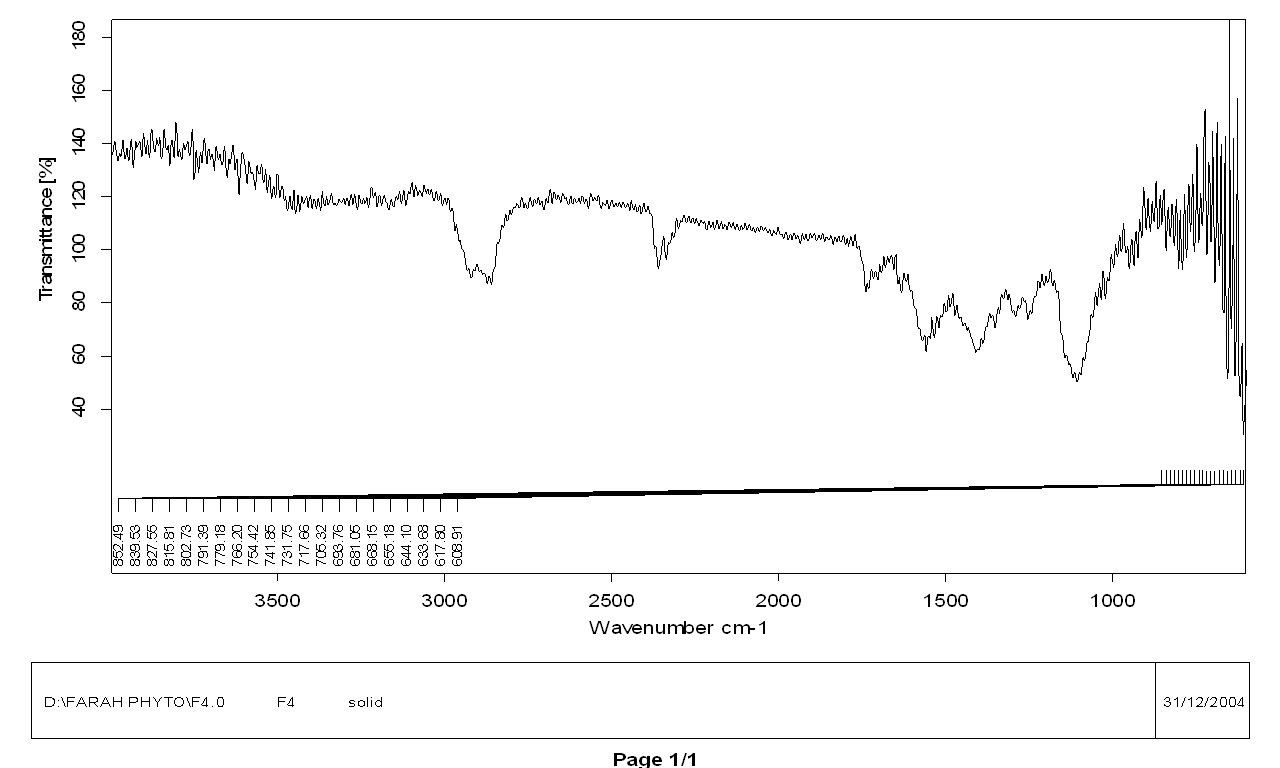


**Figure 10.** FTIR graph of F4.
